# Multi-Channel Fusion Decision-Making Online Detection Network for Surface Defects in Automotive Pipelines Based on Transfer Learning VGG16 Network

**DOI:** 10.3390/s24247914

**Published:** 2024-12-11

**Authors:** Jian Song, Yingzhong Tian, Xiang Wan

**Affiliations:** 1 Shanghai Key Laboratory of Intelligent Manufacturing and Robotics, School of Mechatronic Engineering and Automation, Shanghai University, Shanghai 200444, China; jnsong@shu.edu.cn; 2 Institute of Applied Physics, Jiangxi Academy of Sciences, Nanchang 330000, China

**Keywords:** transfer learning, fusion decision making, fast surface quality screening, surface defect detection

## Abstract

Although approaches for the online surface detection of automotive pipelines exist, low defect area rates, small-sample and long-tailed data, and the difficulty of detection due to the variable morphology of defects are three major problems faced when using such methods. In order to solve these problems, this study combines traditional visual detection methods and deep neural network technology to propose a transfer learning multi-channel fusion decision network without significantly increasing the number of network layers or the structural complexity. Each channel of the network is designed according to the characteristics of different types of defects. Dynamic weights are assigned to achieve decision-level fusion through the use of a matrix of indicators to evaluate the performance of each channel’s recognition ability. In order to improve the detection efficiency and reduce the amount of data transmission and processing, an improved ROI detection algorithm for surface defects is proposed. It can enable the rapid screening of target surfaces for the high-quality and rapid acquisition of surface defect images. On an automotive pipeline surface defect dataset, the detection accuracy of the multi-channel fusion decision network with transfer learning was 97.78% and its detection speed was 153.8 FPS. The experimental results indicate that the multi-channel fusion decision network could simultaneously take into account the needs for real-time detection and accuracy, synthesize the advantages of different network structures, and avoid the limitations of single-channel networks.

## 1. Introduction

### 1.1. The Significance and Development of Automotive Pipeline Surface Defect Detection

With the continuous development of science, technology, computational ability, and Industry 4.0 strategies, a large number of traditional manufacturing industries are facing needs for large-scale production automation and intelligent transformation. This industrial upgrading of China’s manufacturing industry has resulted in higher requirements for product quality, where surface defect detection is an important part of product quality control. To improve the surface defect detection capability on products, particularly in an autonomous and automated manner, intelligent technologies comprise an important part of the development needs to adapt to China’s manufacturing field. Automotive piping, as a connection between the various functional parts of the hose, hard tube, harness, and tubular parts, plays a key role in transferring elements between the various functional parts, such as force, electric current, oil, and other media, thus ensuring that the components achieve their respective functions. Intelligent chassis on high-end automobiles, with components such as air spring suspension systems, and new energy vehicles have a wide range of applications with respect to thermal management. The pipeline’s surface defects directly affect its entire sealing performance, assembly accuracy, and service life, and in serious cases can even affect the safety of the car. Therefore, it is particularly important to detect surface defects to effectively control product quality.

### 1.2. Current Status of Research and Problems Faced

In traditional enterprises, manual visual inspection and machine testing means are generally used. For example, with the help of a magnifying glass, various types of sensors, and other auxiliary instruments, the product surface quality inspection process can be manually completed. However, this approach has great shortcomings, as only part of the surface of the product can be detected under lighting, preventing 360-degree detection of the surface; it can also easily result in visual fatigue, has poor real-time detection performance, and is characterized by leakage and a high misdetection rate. For defect detection on the surface of a product on a roller conveyor, in the best-case scenario, the naked eye can only detect 60% of the defects at a viewing angle of 60–80 degrees; the width of the product cannot be more than 2 m, and the speed of movement cannot be greater than 30 m/s [1]. At present, many enterprises in China still use manual visual inspection-based open-roll sampling methods for product surface inspection, resulting in low efficiency and poor detection results [2]. In recent years, with the development of industrial technology, enterprises have gradually begun to use non-contact non-destructive testing technology represented by machine vision, also called automatic optical inspection (AOI). AOI is a means of achieving automatic inspection through optical system-based imaging, which also has the advantages of high resolution, strong classification, low influence of the environmental electromagnetic field, large working distance, high measurement accuracy, and low cost. Through the online deployment of image acquisition equipment, the acquired image can be transmitted in real time to the monitoring equipment, allowing for the real-time monitoring of the surface state; in this context, the core technical difficulties relate to obtaining accurate and high-quality optical images and processing.

Products moving quickly on a roller conveyor will produce a large number of image data that the system must process, which imposes high requirements for the real-time defect detection algorithm and can lead to the CCD camera images captured having low resolution and lower image quality. Due to the many categories of defect images collected by CCD cameras, in terms of their complex morphology and overlapping boundaries, the collected data form a typical small-sample long-tailed distribution data set. Traditional machine-learning algorithms are often designed as multiple algorithms in series and parallel. Although the recognition accuracy of these algorithms is high, they are time-consuming; affected by the brightness, angle, and edge details of the image; have low generalization ability and robustness; and cannot meet the requirements for the online detection of surface defects [3,4].

After 2010, with the popularization of high-performance computers and improvements in the performance of hardware devices, deep learning has been widely used in many industries, providing new ideas for solving various problems. Based on the idea of deep learning, researchers have conducted in-depth studies for certain problems. Ding et al. (2019) used data mining in the training phase in order to improve the quality of feature information in the region of interest and ameliorate the problems related to small and unbalanced data sets [5]. Tan et al. (2020) fused a transfer learning technique with a convolutional neural network to recognize and classify defects by only fine-tuning the weights, which greatly saves the time needed to train the network [6]. Zhang et al. (2021) proposed a CS-ResNet network model, which adds a cost-sensitive adjustment layer on top of the standard ResNet, assigns larger weights to a few real defects, and utilizes the contribution of minimally weighted equilibrium samples to achieve higher detection accuracy [7]. The idea of other similar methods, such as S-OHEM [8], A-Fast-RCNN [9], Focal Loss [10], and GHM [11], is mainly to use dynamic weights to balance the loss of different samples and alleviate the problem of sample imbalance (i.e., dataset shift). However, allowing the detector to forcibly fit the anomalous samples does not help in the training process and is also prone to causing overfitting-related problems.

With the development of multi-sensor perception technology, scholars have also tried to use multi-modal information and data fusion techniques to address the challenges of small-sample learning. Wanyan Y.Y. et al. (2023) used the idea of active learning to explore multi-modal complementarity in a small-sample learning context for the first time and proposed an adaptive fusion strategy to focus on reliable modalities in few-shot action recognition inference [12]. Hatano M. et al. (2024) proposed a multi-modal cross-domain small-sample learning framework that can effectively utilize visual and non-visual information in small-sample learning situations [13]. Other non-mainstream methods include sample generation [14], data synthesis [15], and so on.

From the above research in the literature, it can be determined that there is a clear trade-off between traditional machine-learning and deep-learning methods. Machine vision algorithms are mature, transparent, and optimized for performance and energy efficiency. While deep learning offers better accuracy and versatility, it also consumes more computational resources. On the other hand, hybrid approaches combine traditional machine vision techniques and deep learning, thus leveraging the benefits of both approaches, and are particularly suitable for high-performance systems that require fast implementation, which is the current mainstream direction of computer vision development.

### 1.3. Scope of Our Work and Contribution

Considering the previous findings, the main contributions associated with the detection network proposed in this study are summarized as follows:

(1) As pipe surface defects have a low area rate in motion and can be seriously interfered with by light and different kinds of noise, as well as CCD camera-based image acquisition, resulting in the effective data resolution and volume being small, the real-time detection of defects poses significant challenges. Therefore, we put forward a rapid surface quality screening algorithm that can assess surface defect images in a timely manner by ignoring the defect-free part of the surface, such that not only is the amount of computation greatly reduced, but the efficiency of detection is also improved, greatly reducing the detection processing time, as well as the transmission and processing volume of data.

(2) Considering the small-sample long-tailed distribution data set problem (i.e., the problem of data sets having a small number of samples and data set shift), this study adopts a transfer learning approach combined with a focal loss class balancing algorithm, where transfer learning solves the problem of few defect samples, while the focal loss class balancing algorithm solves the problem of data set shift, which jointly improves the generalization ability of the model.

(3) Aiming at the problem of detecting defects with variable morphology and subtle defects with small targets, this study designs each channel in the proposed approach according to the characteristics of different types of defects, allowing for the construction of a multi-channel detection network. In order to ensure real-time detection, the network backbone is composed of shallow networks. At the same time, in order to ensure the accuracy of detection, multi-channel decision-level fusion is carried out, such that the final detection results are based on the weighted decision results of each channel. The network simultaneously fulfills the need to balance real-time detection and accuracy, thus avoiding the limitations of single-channel networks.

**Figure 1:**
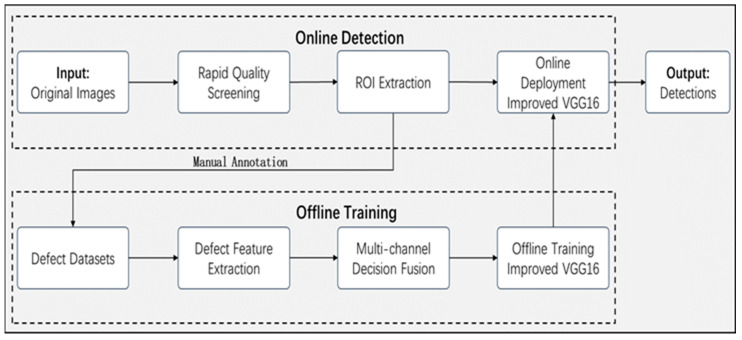
Overview of structural framework for online detection and offline training deployment of multi-channel fusion decision model.

In Figure 1, we present the overview of the automotive pipeline surface defect detection method of this study.

## 2. Related Work

### 2.1. Levels of Fusion

Data fusion allows for the full use of the complementary information between different data sources in order to obtain a more complete and adequate expression, and integrates the information contained in multiple data sources to obtain a consistent and public model output, strengthen the performance of the model, and enhance the interpretability of the fusion model [16]. The existing data fusion techniques can be categorized into three types according to the level of fusion: data-level fusion, feature-level fusion, and decision-level fusion [17].

Data-level fusion, also called pixel-level fusion, is the lowest level of fusion, which is performed after simple preprocessing of the collected raw data, followed by feature extraction being performed on the fused results to draw conclusions. The main advantages of this method include lower loss of data information, the provision of detailed information that cannot be provided by high-level fusion, and the highest accuracy among the current fusion levels.

Feature-level fusion is the intermediate level of fusion, where various feature vectors are first abstracted by a feature extraction algorithm, after which the fusion center completes the fusion processing of the feature vectors. Its advantage is that it achieves data compression, which is conducive to real-time data processing; however, it loses part of the low-level data information and the fusion performance is reduced.

Decision-level fusion, also known as semantic-level fusion, originated in the field of pattern recognition, where different algorithms are used to build recognition models for different data sources, following which the recognition results are combined at the decision level. The purpose of decision-level fusion is to mine and utilize decision-making information from different data sources and to overcome the problems of redundancy, missing data, interference, and uncertainty that may exist in the original data set. This fusion processing method has the advantages of low communication overhead, high interference resistance, and low processing cost.

In order for the fused model to achieve balanced performance in terms of accuracy, data processing speed, and fault tolerance, the existing research on data fusion methods has mainly focused on fusion methods at the feature level and those at the decision level.

### 2.2. Research on Multi-Channel Fusion in Deep Learning

#### 2.2.1. Feature-Level Multi-Channel Fusion in Deep Learning

With the rapid development of deep learning technology and artificial intelligence since 2010, data fusion has fully stepped into the deep-learning stage, with more methods utilizing deep neural networks to learn unified feature representations of data. Deep neural networks are able to automatically learn a multi-layer representation of the data, converting the data into high-level abstract features [18]. With this background, Ngiam et al. (2011) proposed a feature fusion model based on a deep network by coupling a deep auto coder and reconstructing the feature representation through shared features, such that feature fusion could be accomplished through the sharing of intermediate layer features [19]. Dai et al. (2022) proposed an encoder–decoder framework model that uses a multi-feature fusion mechanism, in which all levels of features are fused with each other as a decoder [20]. Zhang et al. (2023) proposed the use of the SimAM parameter-free attention mechanism to evaluate the importance of each neuron in the network and assign corresponding weights [21]. Chen et al. (2024) extracted pre-designed features and high-level semantics for each modal data point before modal fusion, and used an intra-modal attention mechanism to fuse features from different modalities to address the lack of data redundancy [22]. Although academics have made many advances in the field of feature-level fusion of data, there are still some problems with this line of research. In the fusion process, an imbalance among data categories leads to the fusion result being dominated by the category with more data, and these problems are the difficulties that need to be solved urgently in current feature-level data fusion approaches.

#### 2.2.2. Decision-Level Multi-Channel Fusion in Deep Learning

Most of the traditional decision fusion methods use a simple voting method, averaging method, ranking method, or similar, which are simple and easy to model and implement; however, the performance of the fused model is generally poor. Therefore, the current mainstream decision fusion methods mainly use ensemble learning as the guiding idea for fusion. Xiao (2017) designed a combinatorial strategy for integrated learning that combines KNN (K-nearest Neighbor) and SVM (Support Vector Machine) methods to improve the robustness of the model [23]. Zhang et al. (2017) proposed a TOPSIS intuitionistic fuzzy decision-making algorithm based on category similarity weights (CSWBT-IFS) [24]. Chen (2023) constructed a hybrid decision-level fusion based on CNNs (Convolutional Neural Networks) and improved SDAEs (Stacked Denoising Autoencoders) by weighting the two networks based on the weighting determination of the credibility indices [25]. Das R. et al. (2023) implemented a decision fusion mechanism by aggregating the decisions of the three models in a text-sentiment analysis model and computing a score for each sample, fusing the score with the decisions made by the classifier [26]. This type of fusion method does not take into account the characteristics of the data to design the network model, instead relying directly on the results of the model fusion. However, when the network depth is large, this type of fusion leads to poor real-time performance, and considering the volume of data associated with production line equipment, its arithmetic power is insufficient to support deployment and training in this context.

#### 2.2.3. Selection of Fusion Method for This Study

From the above research, it was determined that feature-level fusion methods can fully consider the interactions of features and the correlations between data, the fusion method is simpler, and the losses of information and model performance are smaller; however, the data processing time is longer, and this type of method is more sensitive to the quality of data. Meanwhile, decision-level fusion methods can be regarded as an integrated learning approach considering different data and modal confidence; although the loss of information and model performance requirements are higher, these methods can be well-adapted with respect to the low quality of industrial data and missing data problems. Therefore, the fusion method used in this study is a decision-level fusion method. Table 1 shows the references for feature-level fusion methods and decision-level fusion methods mentioned in this study.

## 3. Rapid Quality Screening and Defect Extraction on Piping Surfaces

### 3.1. Problems Related to Rapid Pipe Surface Quality Screening

In the production process, the moving speed of a pipeline is generally 2–3 m/s, and pipelines presenting surface defects only account for a small part of the total, with the vast majority of the pipelines having no defects. Product surface quality can be monitored in real time by deploying image acquisition equipment in an online manner and transmitting the images captured in real time to the monitoring equipment. However, the performance of the online inspection system can suffer due to background noise, large data volumes, and mismatches between acquisition and transmission speeds during use, resulting in real-time inspection delays and data that cannot be stored. Therefore, in order to efficiently and quickly process a large amount of data, the real-time online inspection system for surface defects is divided into two parts: rapid surface quality screening and defect feature extraction. The rapid surface quality screening approach is designed to detect the presence of suspected defects in images captured in real time, and these images are saved for further processing; meanwhile, images presenting no defects on the surface are ignored. As a large number of defect-free images do not need to be processed, the number of images that need to be manipulated in subsequent processing sessions is greatly reduced, which saves time and improves detection efficiency. It can be seen that the algorithm used for the rapid screening approach has high requirements for simplicity and speed, and a relatively low requirement for accurate localization.

### 3.2. Improved ROI Detection Algorithm for Surface Defects

Blind shooting with the help of a high-resolution CCD camera allows for a large number of images to be obtained in a short time; however, there are great problems related to the storage, labeling, and screening of this massive amount of data. It is inefficient to utilize manual methods to screen and label all the data, and not all of the collected images will contain valid information. For example, in the context of strip surface defect detection in the production process, the strip on the roller conveyor can move more than 10 m/s and the defective area rate is 5% or less, with the vast majority of products presenting no defects on their surface [27]. Therefore, rapid screening of product surfaces to find the ROIs (Regions of Interest) can not only significantly reduce the amount of computation, but also improve the detection efficiency, greatly shorten the detection processing time, and reduce the required amount of data transmission and processing. At present, the most commonly used detection method is the background subtraction method, which generally assumes that defect-free product surface images are more uniform in grayscale and the same batch presents small differences. Therefore, in this study, in order to improve the principle of this algorithm, the grayscale and percentage thresholds, the target image, and the defect-free template image are considered. In particular, the principle of the improved algorithm proposed in this study is to set the gray and percentage thresholds, then compare the average gray value of the target image with that of the defect-free template image in order to determine whether there are defects in the target image. The process is detailed in Table 2.

**Figure 2:**
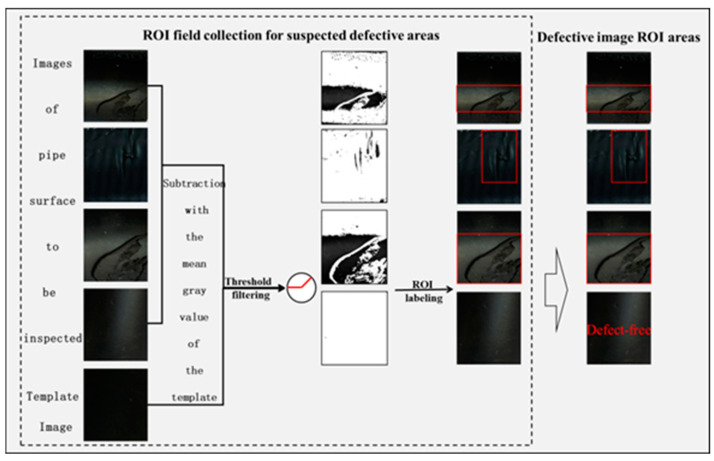
Improved surface defect ROI detection algorithm flow.

As shown in Figure 2, the defect image and the defect-free image are respectively segmented for suspected defects, which are labeled as ROIs, and then filtered to output the ROIs reflecting the image defects. Due to the diverse types of detection objects and defect detection lacking a standard definition, the grayscale and percentage thresholds need to be set according to the actual detection requirements; that is, to meet the needs of the actual data while ensuring that the complexity of the operation is not too high. In addition, for new types of defects, the existence of controversial defects needs to be manually confirmed. At the same time, in actual production, the tester’s experience in the selection of parameters (e.g., to prevent leakage of small defect categories, a smaller threshold is required), the lighting environment, the camera hardware (e.g., image defocusing), and equipment can have impacts on the detection (or misdetection) of defects. In order to prevent this kind of situation, the screened defective images are processed using the Retinex algorithm for surface light equalization, which can help to make the non-formatted data more standardized and unified, thus facilitating the subsequent classification and identification of image defects.

## 4. Surface Defect Detection Network Based on Transfer Learning VGG16 Multi-Channel Fusion Decision Making

### 4.1. Small-Sample Dataset Shift Problem

Standard machine learning algorithms are based on the assumption that there is a sufficient number of samples and a balanced distribution of classes; however, in practice, the data collected for each category have a certain degree of class imbalance (i.e., data set bias). In this type of data set, the amount of data reflecting a specific state (e.g., equipment failure or production line shutdown) is very sparse, leading to a small sample data set. The long-tailed distribution problem, which can seriously affect the effectiveness of a classifier, occurs when the sample categories are very different from each other. When the sample data have a long-tailed distribution, it causes the classifier to be biased, making it more likely to recognize those categories with sufficient sample size and feature diversity. However, the actual effect of the classifier is poor, as it does not learn the features of small samples. To address this type of problem, in traditional machine learning, the expressive power and the ability to fit the training data are enhanced by constructing complex models; however, the training of complex models requires a large amount of training data. If the training data set is too small, the model will suffer from the phenomenon of overfitting (i.e., the model fits the training data very well but generalizes poorly to the test data). In particular, this phenomenon is more significant for deep neural networks [28]. Aiming at this kind of problem, this study proposes an improved VGG16 transfer learning network, combining the “improved ROI region detection algorithm for surface defects” and “class balancing algorithm for Focal Loss” to solve the problem of bias in the small-sample data sets, while also improving the generalization ability of the model.

### 4.2. Multi-Channel Fusion Decision Network Based on Transfer Learning VGG16

#### 4.2.1. Transfer Learning VGG16 Network

Deep-learning-based methods have shown excellent performance in tasks related to surface defect recognition and classification for products. However, the scarcity of defect samples and large feature differences in most industrial products make this type of detection method, which requires training on a large number of defect samples, difficult to apply in actual production. Thus, deep neural networks based on migration learning are widely used. To address this problem, this study transfers the pre-trained weights of the VGG16 network trained in the ImageNet project to the pipeline defect data set used in this study through migration learning in order to complete the pipeline defect transfer learning process. The performance of the model and its errors are closely related, and the accuracy, recall, F1 value, and precision are ideal indicators for evaluating the performance of various network models. In order to reflect the advantages and disadvantages of the model, this study adopts the evaluation indices defined in Equations (1)–(4) in order to evaluate the performance of the model.

(1) $$\mathrm{Accuracy}=\frac{TP+TN}{TP+TN+FP+FN}$$

(2) $$\mathrm{Recall}=\frac{TP}{TP+FN}$$

(3) $$F1=\frac{2TP}{2TP+FP+FN}$$

(4) $$\mathrm{Precision}=\frac{TP}{TP+FP}$$

where TP is the true positive category, FP is the false positive category, FN is the false negative category, and TN is the true negative category.

**Figure 3:**
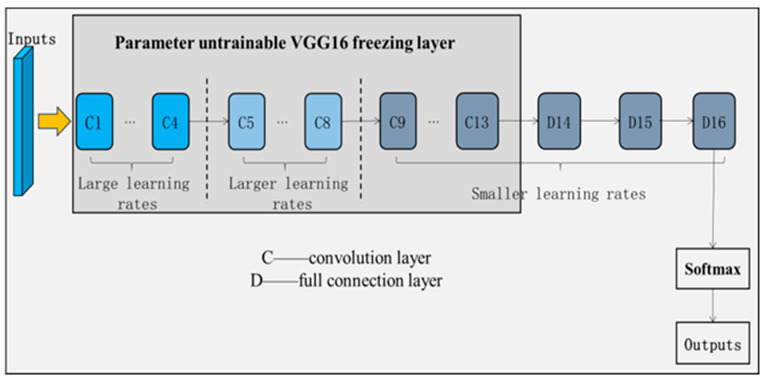
Structure of the VGG16 transfer learning network.

In this study, the VGG16 network is used as the base structure of the CNN network, freezing the first 13 layers without training and using the bottom 3-layer network structure for fine-tuning through training on this study’s data set. The loss function used is the Focal Loss, in order to solve the problem of data bias caused by the imbalance between samples [10]. Finally, the softmax classifier is connected to output the defect categories, thus completing the detection and classification of defects. The learning rate of the network is obtained using a learning rate decay method based on the training step size. As the underlying texture of the pipeline defects is relatively fine, in order to learn the details of its features, the network learning rate needs to be set lower, which can lead to a slow network convergence speed, falling into the local optima, and poor generalization performance. As shown in Figure 3, in order to address the problem of low network learning rate settings, this study improves the learning rate of the network using “hierarchical difference learning rates”; that is, the learning rate of the network at the higher levels is set to a low value, such that it responds to edges and other details and learns carefully. In response, fine-grained geometries are carefully learned. Meanwhile, higher learning rates for the middle and lower levels of the network allow the network to learn image features quickly, thus solving the problem of slow convergence.

#### 4.2.2. Defective Feature Extraction Layers

Compared with traditional machine learning feature extraction algorithms, the key advantage of deep learning feature extraction algorithms mainly lies in their ability to automatically create hidden features from complex data for learning segmentation, rather than completely replacing the feature extraction process used in traditional machine learning, where these features are difficult to manually define. In deep learning, the extraction of features from data mainly relies on the use of a convolution kernel, with different-sized convolution kernels used in accordance with the order and level of collocation, thus constructing different convolutional layers to complete the feature extraction process. By observing the full-field scanning image of the material’s surface, obtained using a light source and a line-scanning CCD camera, it can be found that, when the material surface presents no defects, the reflected light is more consistent with the image signal under a specific field of view. Meanwhile, when there are defects, the light intensity at the sensor location corresponding to a defect will be locally weakened, enhanced, or appear inconsistent with the conventional data on the strip surface, such that various surface defects on the surface of the material can be detected through the changes in the image signal. In order to enable the network to recognize images of different sizes and enhance the robustness of the network, a fully connected layer is not used in the whole network structure. Based on the above problems, three feature extraction modules are designed according to the characteristics of defective images.

(1) Residual maximum average pooling feature extraction module (RMAPM)

**Figure 4:**
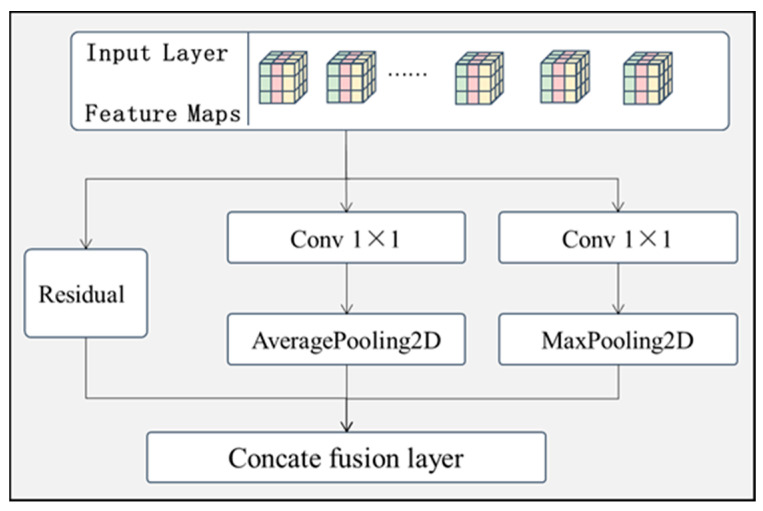
Structure of residual maximum average pooling feature extraction module.

From the surface image of the target to be detected, it can be found that the brightness of the surface defective region is higher or darker than that of the defect-free region. Based on the above problems, a residual maximal average pooling feature extraction layer module was designed to extract features from regions with higher brightness. As shown in Figure 4, this module has three branches.

Branch 1: The original features are not processed, directly bypassing the intermediate layer and fused with the processed feature data via channel connection. The main purpose of this branch is to prevent the model from overfitting due to the presence of too many layers.

Branch 2: The original features are sequentially passed through a convolutional layer of size 1 × 1 and an average pooling layer of size 2 × 2. Branch 2 uses the average pooling layer mainly to filter out the noisy data in the original features and enhance the robustness of the model.

Branch 3: The original features are sequentially passed through a convolutional layer of size 1 × 1 and a maximum pooling layer of size 2 × 2. Branch 3 uses the maximum pooling layer, mainly to extract the foreground features with higher brightness from the original features, in order to better distinguish the background from the foreground in the image to determine the defective regions.

After the original feature data are processed in the three different branches, the different branches are finally connected and fused via the connection fusion layer to provide the fused feature data for the later model.

(2) Residual minimum space pyramid pooling feature extraction module (RMSPPM)

**Figure 5:**
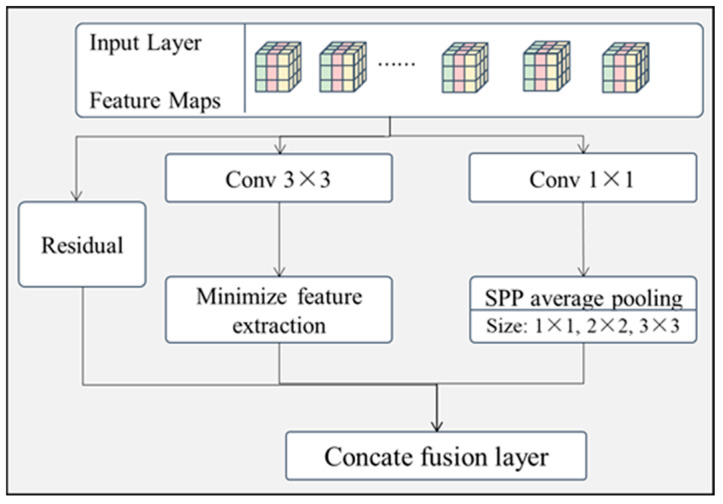
Structure of residual minimum space pyramid pooling feature-extraction module.

As the surface defective regions were found to be darker than the non-defective regions, this subsection continues with the design of a residual-minimization spatial pyramid pooling (SPP) feature extraction module. As shown in Figure 5, this module also has three branches.

Branch 1: The original features are not processed and directly bypass the intermediate layer. The processed feature data are connected and fused via a channel to prevent the model from overfitting problems due to the presence of too many layers.

Branch 2: The original features are sequentially passed through a convolutional layer of size 3 × 3 and then minimized for feature extraction. This branch is mainly for extracting the foreground features with lower brightness from the original features.

Branch 3: The original features are sequentially passed through a convolutional layer of size 1 × 1, then connected to the SPP feature extraction layer. Among them, SPP pooling was selected as the average pooling method, and the window size was selected as 1 × 1, 2 × 2, and 3 × 3, with three scales adapted to different resolutions of image features taking into account both large and small targets, thus facilitating the fusion of features of different scales in the network layer afterward.

The raw feature data are processed in the three different branches and are finally connected and fused via the connection fusion layer.

(3) Residual dilated convolution feature-extraction module (RDCM)

**Figure 6:**
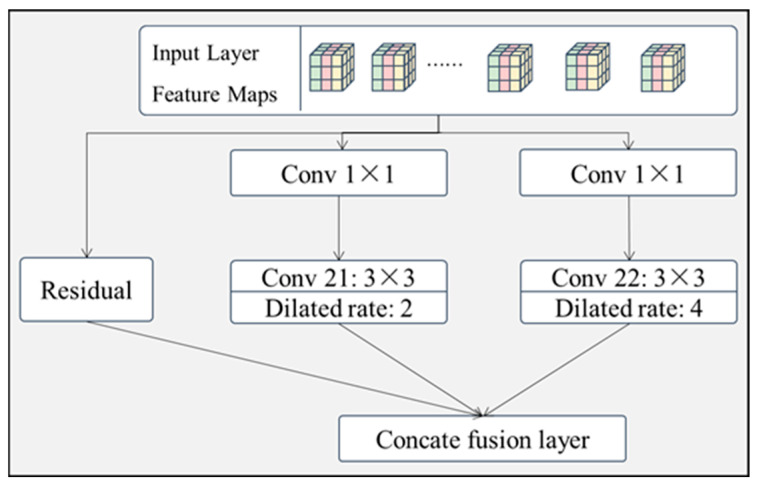
Structure of residual full dilated convolutional feature-extraction module.

As the correlations between input image features are mainly localized and extend from the local to the whole, a residual-connected full null convolutional feature extraction layer is proposed, which combines different scales of null convolutional layers to increase the sensory field of the feature extraction layer without increasing the network parameters, thus completing the extraction of local correlations for different scales of features. As shown in Figure 6, the module has three branches.

Branch 1: The original features are not processed, directly bypassing the intermediate layer, and fused with the processed feature data via channel connection. This branch mainly prevents the model from overfitting due to the presence of too many layers.

Branch 2: The original features are sequentially convolved by a 1 × 1 convolution, then connected to a 3 × 3 convolution layer, where the expansion rate of convolution kernel 21 is set to 2.

Branch 3: The original features are sequentially passed through a convolution of size 1 × 1, then connected to 3 × 3 convolution layers, where the expansion rate of convolution kernel 22 is set to 4.

The raw feature data are processed in three different branches and finally fused via a connection fusion layer connected by branches, where a convolutional layer of size 1 × 1 enriches the raw features by increasing the number of channels of the raw input.

#### 4.2.3. Multi-Channel Fusion Decision Network

The recognition accuracy of different structural network models for various types of data is inconsistent (i.e., a given network model will be stronger for some categories of data, but weaker for other categories of data). The general approach to this problem is to target the construction of deeper and more complex network structures, or use specially designed network layers to improve the performance of the model. These complex networks amplify the performance difference between the architectures and effectively improve the feature extraction ability of the network. For example, the ResNet network, represented by the skip-connection technique, solves the gradient vanishing problem to a certain extent and obtains a level of performance improvement [29,30]. This kind of structure causes the network depth to increase dramatically, from 5 layers in LeNet and 16 layers in VGGNet to more than 100 layers in ResNet. Standard deep-learning approaches gradually lose plasticity as learning continues, but as the network deepens, a deep network does not necessarily learn better than a shallower network [31]. As the network depth increases, although it brings a certain level of performance improvement, it also brings problems related to the complexity of the model’s structure, making it difficult to deploy and train the model. In order to address the difficulty of deployment and training brought about by the complex model structure, this study uses the multi-channel fusion method to integrate the information of multiple channels, realize the complementary advantages, and make up for the lack of performance of a single model, to a certain extent. This also means that the depth and complexity of the single-model network structure can be effectively reduced, alleviating the need to use specially designed network layers to improve the performance of a single model, as well as greatly reducing the difficulty of designing the network structure and deploying the model for training. The multi-channel fusion decision approach used in this study evaluates the performances of different channels in terms of data learning and recognition through the evaluation metrics detailed in the previous section, and then assigns dynamic weights to each channel to achieve decision-level fusion.

Defining n channels and m evaluation indices, the whole network obtains an n×m matrix of evaluation indices, where each channel generates m evaluation indices x_ij_ from the final feature extraction layer as the performance evaluation criteria. Finally, the dynamic weights of each channel are calculated to achieve the dynamic decision fusion of the weights, and classification of the data samples into K classes is performed. The accuracy, recall, F1 value, and precision were selected as evaluation indices in this study. The dynamic weight calculation process for each channel is described as follows.

① Indicator attribute normalization processing. Low- and medium-performance indicators are converted into high-performance indicators x′_ij_. That is, the data are normalized, such that the larger the value of these indicators, the better the performance of the model. The conversion method is shown in Equation (5).

(5) $$x_{ij}^{\prime }=\left\{\begin{array}{c} x_{ij}\;\;\;\;\;\;\;\;\mathrm{High}-\mathrm{performance}\;\mathrm{indicator}\;\;\;\;\\ 1/x_{ij}\;\;\;\;\;\;\;\mathrm{Low}-\mathrm{performance}\;\mathrm{indicator}\;\;\;\;\\ M/(M+\left|x_{ij}-M\right|)\;\;\mathrm{Medium}-\mathrm{performance}\;\mathrm{indicator}\;\;\;\; \end{array}\right.$$

where i ∈ [1, n] and j ∈ [1, m].

② Combination of normalized data.


(6) $$Z_{ij}=\left\{\begin{array}{c} \frac{x_{ij}}{\sqrt{\sum_{i=1}^{n}{\left(x_{ij}\right)}^{2}}}\;\;\mathrm{High}-\mathrm{performance}\;\mathrm{indicator}\;\;\;\;\\ \frac{x_{ij}^{\prime }}{\sqrt{\sum_{i=1}^{n}{\left(x_{ij}^{\prime }\right)}^{2}}}\;\;\mathrm{Other}\;\mathrm{performance}\;\mathrm{indicator} \end{array}\right.$$

This results in the normalized matrix Z.


(7) $$Z=\left[\begin{array}{cccc} z_{11} & z_{12} & \ldots & z_{1m}\\ z_{21} & z_{22} & \ldots & z_{2m}\\ \ldots & \ldots & \ldots & \ldots \\ z_{n1} & z_{n2} & \ldots & z_{nm} \end{array}\right]$$

③ Determine the optimal and worst solutions. The optimal solution Z^+^ consists of the maximum value in each column of Z. Similarly, the worst solution Z^-^ consists of the minimum value in each column of Z.

④ Calculate the distances D^+^_i_ and D^−^_i_ from Z^+^ and Z^−^ for each channel.


(8) $$D_{i}^{+}=\sqrt{\sum_{i=1}^{m}{\left(\max Z_{ij}-Z_{ij}\right)}^{2}}$$

(9) $$D_{i}^{-}=\sqrt{\sum_{i=1}^{m}{\left(\min Z_{ij}-Z_{ij}\right)}^{2}}$$

⑤ Calculate the proximity C_i_ of each evaluation channel to the optimal solution.

(10) $$C_{i}=\frac{D_{i}^{-}}{D_{i}^{+}+D_{i}^{-}}$$

where 0 ≦ C_i_ ≦ 1 and C_i_→1, indicating better performance of a channel.

⑥ Calculate the weight W_i_ for each channel, in accordance with Equation (11).


(11) $$W_{i}=\frac{C_{i}}{\sum_{i=1}^{n}C_{i}}$$

⑦ k is the prediction category of each channel for the Ith sample and $P_{k}^{i}(I)$ is the prediction confidence value of the ith channel for the Ith sample.

(12) $$Vote(k_{I})=\underset{k}{\arg\max}[\sum_{I=1}^{K}P_{k}^{i}(I)*W_{i}]$$

where k ∈ [1, K], and the sum of the weighted values of each channel’s vote for the Ith sample is the largest for the final prediction category k_I_.

As the VGG neural network (Very Deep Convolutional Networks designed by the Visual Geometry Group) is a classical and simple linearly structured network, the network pre-training recognition accuracy is high, and it is convenient to modify its network structure. Therefore, the VGG16 network was chosen as the backbone network in this study. The common feature of the VGG neural network structure is that several convolutional layer modules are connected to three fully connected layers and finally a softmax layer is used to recognize and classify the data. As the three fully connected layers occupy most of the parameters in the network structure, this greatly slows down network training, reduces the recognition efficiency, and increases the risk of overfitting; furthermore, the network can only recognize images of uniform size, which greatly limits the diversity of the feature data extracted by the network.

**Figure 7:**
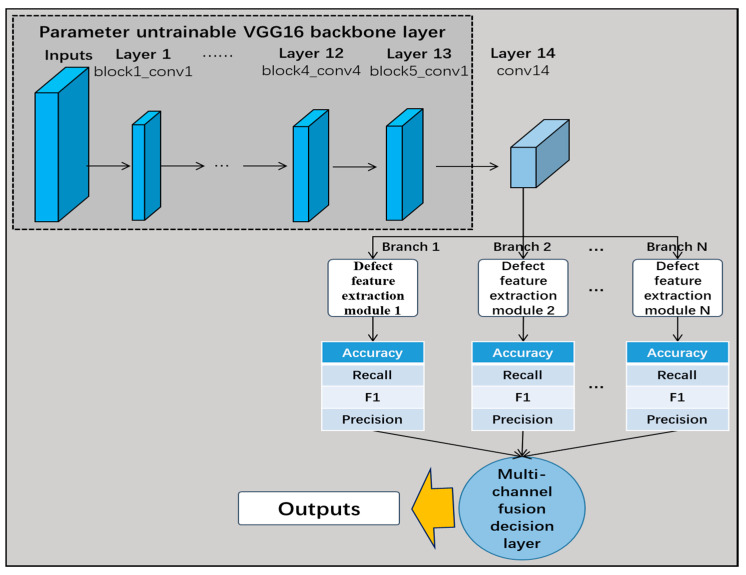
Multi-channel fusion decision network based on migration learning VGG16.

As shown in Figure 7, this study proposes the multi-channel fusion decision network based on the transfer learning VGG16 model, which removes the three fully connected layers when using the VGG16 network as the backbone of the pre-trained network. While the first 13 frozen layers are still untrainable, the 3 fully connected layers are replaced with 1 new convolutional layer. Each branch of the 14th output of these layers is connected to the defective feature-extraction module. Finally, the multi-channel fusion decision layer calculates the weights of each channel to make dynamic fusion decisions. The performance of the fused shallow network model is better than that of the deep model with a simple deepening of the number of layers, and the fusion results are insensitive to the parameters of the model, robust, and have better real-time performance.

## 5. Online Detection of Surface Defects on Automotive Piping

### 5.1. Experimental Environment, Data Preparation and Validation Process

#### 5.1.1. Experimental Environments

**Figure 8:**
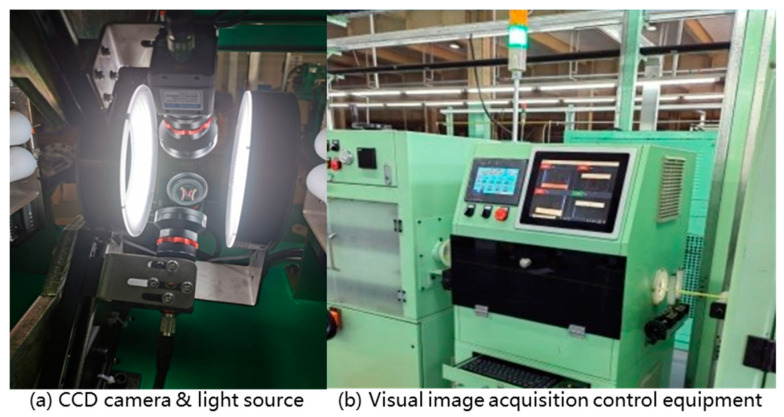
Pipe surface image acquisition system.

As shown in Figure 8, the experimental platform comprised four Hikvision CCD cameras (Hangzhou, Zhejiang, China) with fixed-frequency shooting. The illumination light source for the two middle apertures of the ring was a mounted diffuse reflection shadowless light source, issued from the light source openings through the billet to be detected. In the pipe image acquisition system, the four cameras were responsible for acquiring images with 90 degrees, achieivng 360 degree detection of the circumference of the surface of the pipe billet without any dead angle. Each CCD camera lens had a front distance from the billet of 100 mm, the distance between the two light sources was 250 mm, and the system could detect the diameter of the billet specifications for φ40 to φ120 mm.

The algorithm-running platform for the experiments in this study was a Windows 10 operating system personal computer with an I7-8500 processor, NVIDIA GTX3070 graphics card, 12 G of video memory, 64 G of RAM, and a hard disk capacity of 1 T. The algorithm programming was implemented using the Python 3.8 programming language, including the Keras 2.3.0 and TensorFlow 1.15 frameworks.

#### 5.1.2. Data Preparation

**Figure 9:**
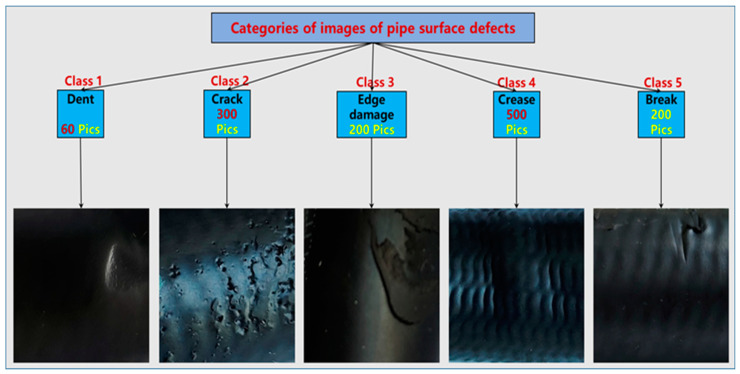
Pipeline defect category.

The experimental data in this study were image data of pipe surface defects collected from an automotive pipeline production line, comprising five categories—dent (60), crack (300), edge damage (200), crease (500), and break (200)—for a total of 1260 images, where each image was sized 200 × 200 pixels. The images in the data set were grayscale images in .jpg format, 80% of the data were used for training, and performance testing was conducted on the remaining 20% of the data. Figure 9 shows examples of the surface defect category categories.

#### 5.1.3. Experimental Verification Process

The pipeline surface defect detection technology presented in this study is a combination of traditional algorithms and deep learning classification methods, in which the traditional algorithms quickly screen the surface quality, quickly exclude the defect-free images, and find the defective ROI, improving the real-time detection performance. The deep-learning algorithm uses the defect feature-extraction layer designed in this study, combined with improved transfer learning on the VGG16 network and multi-channel fusion decision-making, thus achieving defect detection of dents, cracks, damage, and other variable morphological defects, thus enhancing the generalization and detection accuracy of the model. The specific experimental process was as follows: fast surface quality screening with traditional algorithms, ROI detection of surface defects using traditional algorithms, and deep-learning-based defect detection and classification. For performance verification of the improved network proposed in this study, the improved transfer learning VGG16 network was connected to the three defect feature-extraction layers designed in this study and the multi-channel fusion decision-making network and a performance comparison test was carried out on the pipeline defect data set collected in this study to finally determine the network structure with the best performance.

### 5.2. Transfer Learning VGG16 Online Detection Network

#### 5.2.1. Transfer Learning VGG16 Network

**Figure 10:**
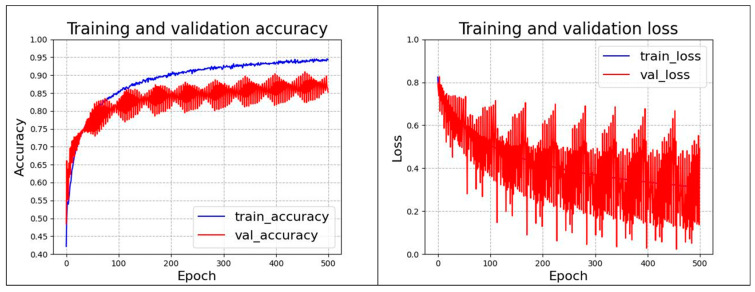
Network accuracy and loss error.

In this paper, we complete the pipeline defect transfer learning by transferring the pre-training weights of the VGG16 network trained by the ImageNet project to the pipeline defect dataset in this study. The VGG16 transfer learning network freezes the first 13 layers without training and uses the remaining 3 fully connected layers for training. The network layers were set with learning rates of 10^−4^, 10^−5^, and 10^−6^ for different layer regions with the 1:1:2 rule, a decay momentum of 0.9, and training step size of 500, respectively. For the dataset shift problem in a dataset, a class balancing algorithm using focal loss was used to solve the problem [10]. The network accuracy and loss error are shown in Figure 10, which shows that the network had poor accuracy and convergence. Particularly on the test dataset, the performance of the network fluctuated a lot.

**Figure 11:**
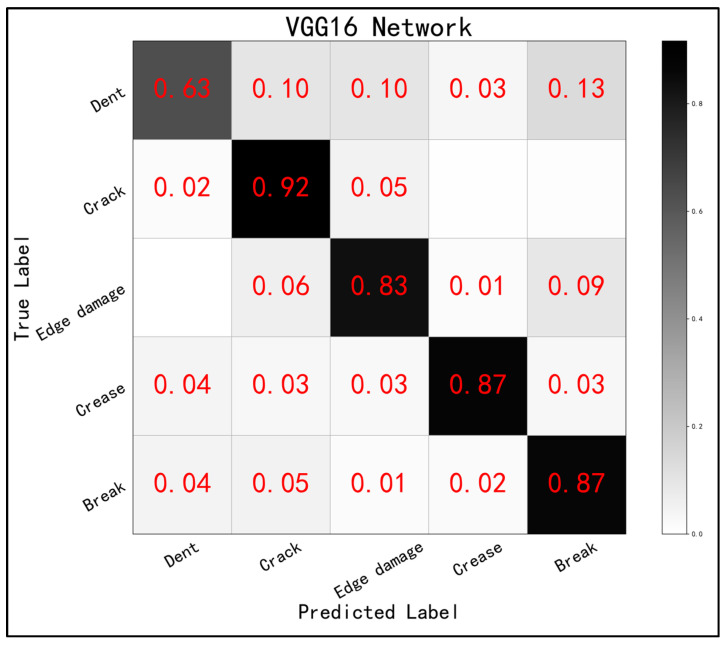
Confusion matrix for network.

The confusion matrix of the network is shown in Figure 11, from which it can be seen that the overall recognition rate of the network for each category of defects was low. This was due to the inconsistency in the amount of image data, the size of the defective region area, and the difficulty of recognition for each class in this dataset, indicating evident problems related to the long-tailed distribution and small samples. Although the focal loss class balancing algorithm can alleviate such problems, it also requires a specially designed network structure, which is the focus of this study.

The common feature of the VGG neural network structure is that several convolutional layer modules are connected to three fully connected layers, and finally the data are recognized and classified by a softmax layer. As the three fully connected layers occupy most of the parameters in the network structure, this will greatly slow down the network training and recognition efficiency, increase the risk of overfitting, and force the network to only recognize images of uniform size, which greatly limits the diversity of features extracted by the network. Therefore, in this study, we subsequently improve the transfer learning VGG16 network by removing its three fully connected layers.

#### 5.2.2. Improved Transfer Learning VGG16 Network and Performance Comparison

**Figure 12:**
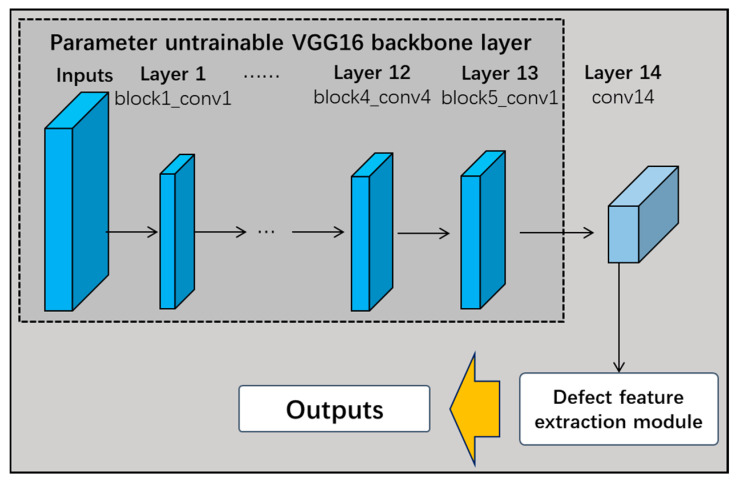
Improved transfer learning VGG16 network structure diagram.

The VGG16 network was taken as the backbone of the pre-trained network, removing its three fully connected layers and replacing them with one convolutional layer; meanwhile, the first 13 frozen layers were still retained. The output of the 14th layer was connected to the three defect feature extraction modules designed in this study for recognition (RMAPM, RMSPPM, and RDCM). In Figure 12, the structure of the improved transfer learning VGG16 network is illustrated.

**Figure 13:**
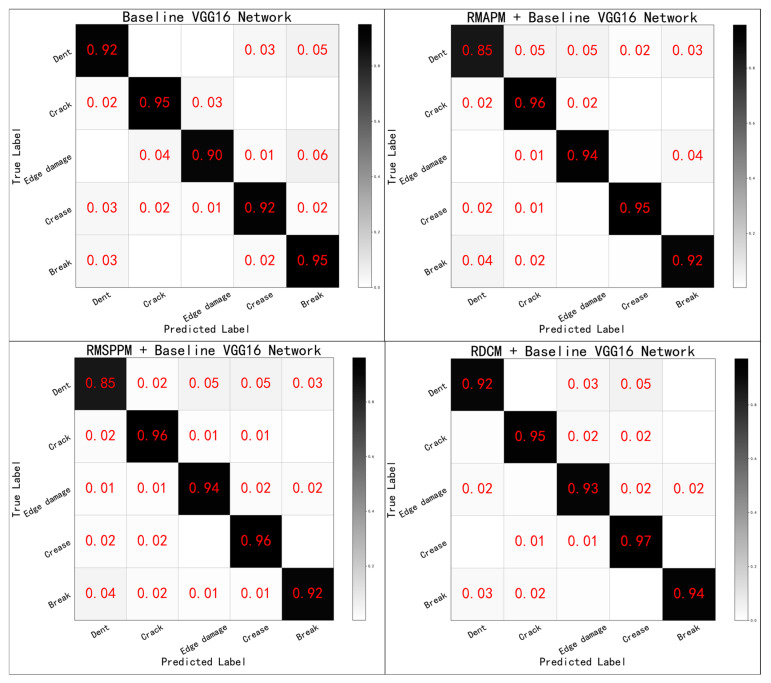
Confusion matrix performance comparison for each network.

Figure 13 shows the network without adding the various modules as the baseline VGG16 network and compares its performance difference with those of the networks including each defective feature extraction module. As the improved network structure did not use the fully connected layer, it could recognize images of different sizes, which not only enhanced the robustness and accuracy of the network, but also effectively reduced the parameters of the network and accelerated the convergence speed.

As shown in Table 3, the performance of each network was compared in an ablation experiment. The RMAPM module, the RMSPP module, and the RDCM module had improved performance for the baseline VGG16 network. Specifically, the RDCM module combined different scales of null convolutional layers to increase the sensory field of the feature-extraction layer and provide the most evident performance improvement for the baseline VGG16 network. It can be seen that, although the performance of the network with the addition of each defect feature-extraction module (baseline VGG16 network) was improved, the ability of each defect feature-extraction module to recognize the various defects was different. Some types of defects, such as dents, are also very difficult to recognize by the human eye and are not suitable for training deep neural networks. If the ability of each module is fused for recognition, then there is some room for improvement in the overall performance of the network.

### 5.3. Transfer Learning VGG16 Multi-Channel Fusion Decision-Making Online Detection Network

#### 5.3.1. Multi-Channel Fusion Decision-Making for Online Detection Networks

**Figure 14:**
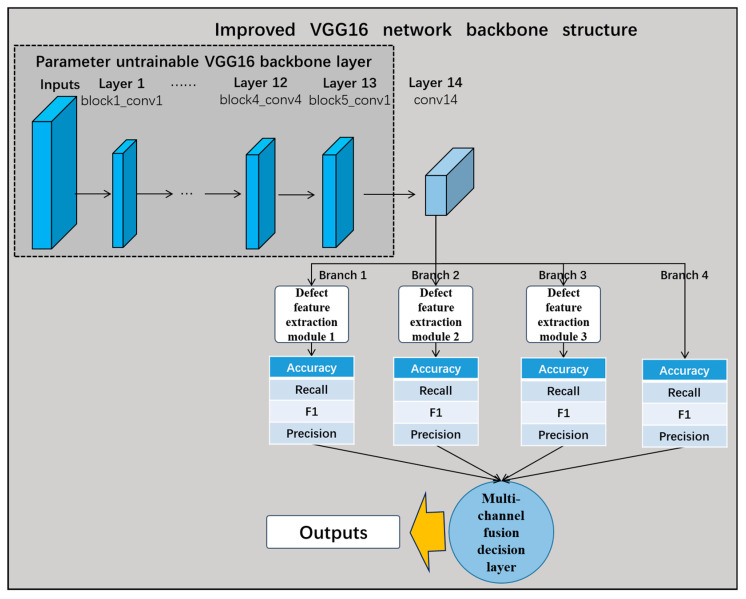
Multi-channel fusion decision network based on transfer learning VGG16.

As shown in Figure 14, to address the problem associated with the performance of a single network for the recognition of different classes of defect, as discussed in the previous subsection, this study proposes the multi-channel fusion decision network based on transfer learning VGG16. The VGG16 network was selected as the backbone of the pre-trained network, removing its three fully connected layers and replacing them with a convolutional layer, while the first 13 frozen layers were retained. The three branches of the 14th layer’s output were connected to three defect feature extraction modules, while branch 4 was not connected to the feature extraction module. Finally, the multi-channel fusion decision layer calculated the weights of each channel and made a dynamic fusion-based decision.

**Figure 15:**
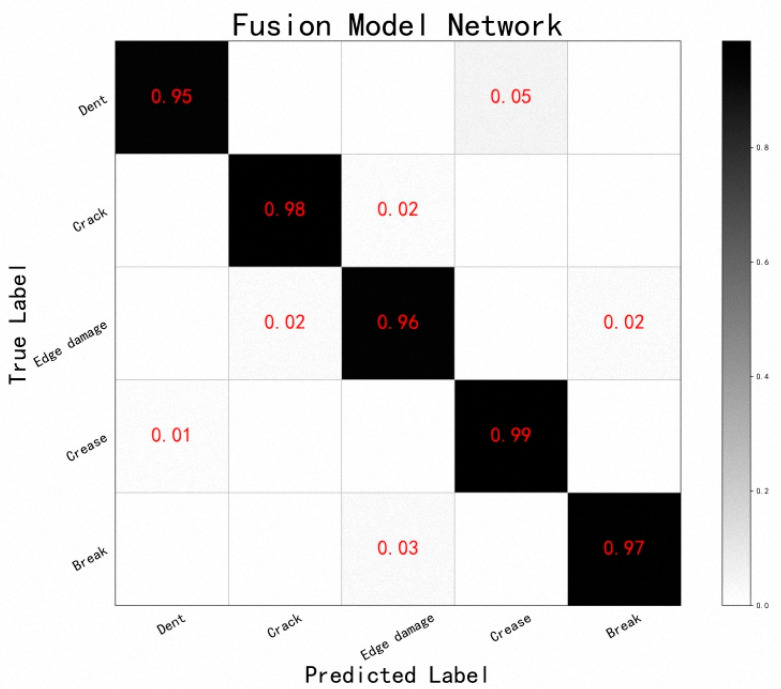
Confusion matrix for fused network.

In Figure 15, the confusion matrix of the network with four-branch fusion is shown. It can be seen that the performance of the fused network significantly improved relative to the performance of a single network. Thus, the use of data-fusion methods to integrate the information of different network modules to achieve complementary advantages can compensate for the lack of performance of a single network to a certain extent.

#### 5.3.2. Confusion Matrix for Fused Networks

In Table 4, the network performance before and after fusion is compared. It is clear that the fusion network could dynamically assign weights according to the performance of each branch and make full use of the advantages of each feature-extraction layer to effectively improve the accuracy and credibility of the fusion decision. The performance of the fused network was greatly improved, compared with the performance of each single network. This also means that the fused network could effectively reduce the dependence on the depth and complexity of the single model network structure, greatly reducing the difficulty of designing the network structure and deploying the model for training. The performance of the fused shallow network model was better than that of the deep model with simply deeper layers, and the fusion result was insensitive to the parameters of the model, is robust, and had better real-time performance.

To further verify the performance of the algorithm proposed in this study, we tested and compared various algorithms on the automotive pipeline surface defect data. As can be seen in Table 5, traditional algorithms are faster than deep networks, in terms of recognition speed, but have low accuracy and poor utility. The fusion network model in this paper not only achieves high accuracy and recognition speed, but also prevents the problems of non-convergence and overfitting.

In summary, fusion network’s advantages and disadvantages can be concluded.

- The fusion network can effectively improve the detection accuracy of the lightweight level network and meet the demand of real-time detection without significantly increasing the depth and structural improvement of the network;
- Fusion network combine multiple network branches, so it takes more time than its counterparts in terms of the inference time;
- A very deep network is not necessarily required for the online real-time defect classification task and the detection accuracy, speed, and complexity of the network need to be balanced.

As the fusion decision network is a lightweight level network, there was still a gap between the proposed network model and the current state-of-the-art YOLO algorithm and its improved algorithm (state-of-the-art) in terms of detection accuracy and detection speed [34,35,36]. Therefore, we will continue to pay attention to this issue in subsequent research in order to further balance the detection accuracy, detection speed, and network lightweight.

## 6. Conclusions

In this study, a multi-channel fusion decision network based on transfer learning was proposed to evaluate the performance of each channel’s recognition ability by evaluating the index matrix and assigning dynamic weights to realize decision-level fusion, yielding high accuracy and good real-time performance. The fusion model has a simple structure, few network layers, and low model memory usage, which makes it a lightweight network that is suitable for industrial deployment. The multi-channel fusion decision network with transfer learning achieved 97.78% detection accuracy and 153.8 FPS detection speed on the automotive pipeline surface defect dataset. The experimental results demonstrated that the proposed algorithm did not require data preprocessing, as raw data (images) could be directly input into the network; furthermore, the model remained robust and accurate on small-sample data sets, was insensitive to changes in the parameter settings, and could be practically and flexible deployed, making it suitable for the implementation of approaches promoting the effective detection and recognition of various types of surface defects. The defective feature extraction layers (i.e., RMAPM module, RMSPP module, and RDCM module) and fusion decision network proposed in this study are strongly portable and can be combined with the feature pyramid network or various improved YOLO networks’ detection heads to further improve the performance of the network. This will also be the following research direction of this paper.

## Figures and Tables

**Table 1:** Feature-level fusion methods compared with decision-level fusion methods.

Literature	Methods	Fusion Level
2011, [19]	Reconstructing feature representations by coupled deep auto coders	Feature-level
2022, [20]	Encoder–decoder framework model	Feature-level
2023, [21]	SimAM parameter-free attention mechanism	Feature-level
2024, [22]	Intra-modal attention mechanism to fuse features	Feature-level
2017, [23]	KNN + SVM	Decision-level
2017, [24]	CSWBT-IFS	Decision-level
2023, [25]	CNN + stacked denoising autoencoders	Decision-level
2023, [26]	Computing a score for each sample, fusing the score with the decisions made by the classifier	Decision-level

**Table 2:** Improved defective ROI detection algorithm pseudo-code.

**Algorithm 1** Improved ROI detection algorithm
**Input:** Original images, template image
**Output:** Defect ROI
**Step 1. Calculate Initial Values**
//P_ROI_ can be set to a value or to 100% of the full image area. **Set** thresholds P_max_, P_min_, P_ROI_. //Calculate the average value of the template image, template_average. template_average = average(template image) **Step 2. original_images subtract from template_average** //If template image is complex and not suitable for calculating average value, original images can be subtracted directly from the template image. **for** original_images **in** target_path **do** result = original_images − template_average result[(result < P_min_) | (result > P_max_)] = 0 return result **Step 3. Find ROI** //Find contours in the binary image Contours = cv2.findContours(binary) //Filter contours based on area rois = [cnt for cnt in contours if cv2.contourArea(cnt) > P_ROI_] //Draw contours on the original image and save cv2.drawContours(original_image, rois) //Find the minimum bounding rectangle for all ROIs all_rois = concatenate(rois) cv2.boundingRect(all_rois) //Draw the minimum bounding rectangle cv2.rectangle(original_image)

**Step 4. Output**
Get Defect ROI

**Table 3:** Results of ablation experiment.

	Models	Baseline VGG16	Baseline VGG16 + RMAPM	Baseline VGG16 + RMSPP	Baseline VGG16 + RDCM
Metrics					
Ablation Module		14 CNN √	14 CNN √	14 CNN √	14 CNN √
		RMAPM ×	RMAPM √	RMAPM ×	RMAPM ×
		RMSPP ×	RMSPP ×	RMSPP √	RMSPP ×
		RDCM ×	RDCM ×	RDCM ×	RDCM √
Accuracy		0.9278	0.9405	0.9444	0.9532
Recall		0.9278	0.9405	0.9444	0.9532
F1		0.9294	0.9420	0.9457	0.9535
P_Dent_		0.6707	0.6623	0.6711	0.7209
P_Crack_		0.9498	0.9472	0.9503	0.9564
P_Edge damage_		0.9227	0.9303	0.9495	0.9118
P_Crease_		0.9788	0.9876	0.9757	0.9759
P_Break_		0.8879	0.9337	0.9581	0.9793

**Table 4:** Comparison of network performance before and after fusion of branches.

Branch		Branch1	Branch2	Branch3	Branch4	Fusion Layer
	Models	RMAPM + VGG16	RMSPPM + VGG16	RDCM + VGG16	Baseline VGG16	Fusion Model
Metrics						
Accuracy		0.94047	0.9444	0.95317	0.9278	0.9778
Recall		0.9405	0.9444	0.9532	0.9278	0.9782
F1		0.942	0.9457	0.9535	0.9294	0.9779
P_Dent_		0.6623	0.6711	0.7209	0.6707	0.8906
P_Crack_		0.9472	0.9503	0.9564	0.9498	0.9866
P_Edge damage_		0.9303	0.9495	0.9118	0.9227	0.9505
P_Crease_		0.9876	0.9757	0.9759	0.9788	0.9940
P_Break_		0.9337	0.9581	0.9793	0.8879	0.9799

**Table 5:** Various algorithms tested on surface defect data.

Type	Algorithm	Accuracy	Top1@mAP0.5	Time per Image	Memory Size of Weights
Traditional Algorithms	KNN	83.45%	-	0.0020 s 500.0 FPS	-
	Gradient Boosting	86.32%	-	0.0032 s 312.5 FPS	-
Deep-Learning Algorithms	PLCNN [32], 2020	94.71%	94.13%	0.0053 s 188.7 FPS	58.0 MB
	ResNet50 + MFN [33], 2020	96.46%	95.64%	0.0069 s 144.9 FPS	62.1 MB
	YOLOX-m [34], 2021	98.03%	97.51%	0.0075 s 133.3 FPS	186.8 MB
	Scaled-YOLOv4-CSP [35], 2021	98.42%	98.12%	0.0053 s 188.7 FPS	213.5 MB
	YOLOv7-E6 [36], 2023	99.13%	98.76%	0.0046 s 217.4 FPS	192.1 MB
	Ours’	97.78%	96.03%	0.0065 s 153.8 FPS	56.2 MB

## Data Availability

The data presented in this study are available on request from the corresponding author. The data are not publicly available.
